# High-density lipoproteins downregulate CCL2 production in human fibroblast-like synoviocytes stimulated by urate crystals

**DOI:** 10.1186/ar2930

**Published:** 2010-02-11

**Authors:** Anna Scanu, Francesca Oliviero, Lyssia Gruaz, Paolo Sfriso, Assunta Pozzuoli, Federica Frezzato, Carlo Agostini, Danielle Burger, Leonardo Punzi

**Affiliations:** 1 Department of Clinical and Experimental Medicine, University of Padova, Via Giustiniani 2, 35128 Padova, Italy; 2 Division of Immunology and Allergy, Hans Wilsdorf Laboratory, IARG Department of Internal Medicine, University Hospital and Faculty of Medicine, University of Geneva, 4 rue Gabrielle-Perret-Gentil, CH-1211 Geneva 14, Switzerland; 3 Department of Medical and Surgical Specialties, Orthopaedic Clinic, University of Padova, via Giustiniani 3, 35128 Padova, Italy

## Abstract

**Introduction:**

To investigate whether monosodium urate (MSU) crystals induce the production of CCL2 (monocyte chemoattractant protein-1; MCP-1) in human fibroblast-like synoviocytes (FLS) and whether this mechanism would be affected by high-density lipoproteins (HDL).

**Methods:**

Human FLS isolated from synovial tissue explants were stimulated with MSU crystals (0.01 to 0.5 mg/ml) or interleukin (IL)-1β (10 pg/ml) in the presence or absence of HDL (50 and 100 μg/ml). The production and expression of CCL2 was evaluated with ELISA, confocal microscopy, immunofluorescence microscopy, chemotaxis assay, and real-time quantitative PCR.

**Results:**

Exposure of FLS to MSU crystals induced CCL2 accumulation in culture medium in a dose- and time-dependent manner, reaching a plateau at 50 to 75 μg/ml MSU crystals and 20 to 24 hours. Although low, the induced CCL2 levels were sufficient to trigger mononuclear cell migration. In resting FLS, CCL2 was localized in small cytoplasmic vesicles whose number diminished with MSU crystal stimulation. Concomitantly, MSU crystals triggered the induction of CCL2 mRNA expression. All these processes were inhibited by HDL, which cause a 50% decrease in CCL2 mRNA levels and a dose-dependent inhibition of the release of CCL2. Similar results were obtained when FLS were pretreated with HDL and washed before activation by MSU crystals or IL-1β, suggesting a direct effect of HDL on the FLS activation state.

**Conclusions:**

The present results demonstrate that MSU crystals induce FLS to release CCL2 that is stored in vesicles in resting conditions. This mechanism is inhibited by HDL, which may limit the inflammatory process by diminishing CCL2 production and, in turn, monocytes/macrophages recruitment in joints. This study confirms the antiinflammatory functions of HDL, which might play a part in the limitation of acute gout attack.

## Introduction

CCL2 (monocyte chemoattractant protein-1; MCP-1), a member of the C-C chemokine family, is a major monocyte chemoattractant [1]. CCL2 production is inducible in various types of cells, including synoviocytes [2,3]. *In vivo *studies suggest that CCL2 attracts monocytes to sites of inflammation in a variety of pathologic conditions, including atherosclerosis [4,5], pulmonary fibrosis and granulomatous lung disease [6], and degenerative and inflammatory arthropathies, including gout [7-9]. Gout is a consequence of elevated serum urate levels that lead to deposition of monosodium urate (MSU) crystals in joints, causing an acute inflammatory response [10]. MSU crystals are indeed potent inducers of inflammation, as demonstrated *in vivo*. When injected into the peritoneum or in the air pouch of animal models, MSU crystals induce an inflammatory response characterized by a cellular infiltrate rich in neutrophils and the production of proinflammatory cytokines, as well as other inflammatory mediators, including CCL2 [11-13]. CCL2 has long been associated with crystal inflammation. Elevated levels of CCL2 were measured in synovial fluid of gout patients [14]. Besides, in gouty arthritis models, intraarticular injection of MSU crystals induces the rapid release of CCL2 within 1 hour after injection, reaching a maximum at 2 to 4 hours [7]. Thus, CCL2 might be involved in the recruitment of monocytes/macrophages at the site of inflammation.

Once infiltrated in the joints, MSU crystals trigger monocytes/macrophages to produce IL-1β, a mechanism highly relevant to gout, the acute form of which is effectively treated with the recombinant form of IL-1-receptor antagonist, a specific IL-1 inhibitor [15,16]. Although the presence of MSU crystal-specific receptor at the cell surface is unlikely, MSU crystals might stimulate cells through membrane lipid alteration [17].

By secreting CCL2, activated resident synoviocytes may display the ability to recruit monocytes into the joints and, in turn, to set in the inflammatory response that underlies the acute attack of gout. In most cases, the acute attack is self-limited by processes that remain largely unknown [18]. However, a number of plasma proteins and lipoproteins that suppress the MSU crystals deleterious effects have been identified in synovial fluids. Among them, apolipoprotein (apo) B and apo E inhibit crystal-induced neutrophil stimulation by binding to the surface of crystals [19,20]. In addition, low-density lipoproteins (LDL) and high-density lipoproteins (HDL) strongly inhibit calcium and MSU crystal-induced neutrophil cytolysis [21], and LDL contribute to the resolution of acute inflammatory attack induced by calcium crystals in the rat air-pouch model [22]. Recently, we demonstrated that HDL-associated apo A-I can exert antiinflammatory effects through the inhibition of cytokine production in monocytes/macrophages on contact with stimulated T cells or with stimulated T cell-derived microparticles [23,24]. Together, these studies suggest that lipoproteins may act at several levels to dampen inflammation.

Because MSU crystals increase CCL2 expression in vascular smooth muscle and epithelial cells [25,26], this study was undertaken to assess whether MSU crystals might display similar activity toward fibroblast-like synoviocytes (FLS), and whether this activity might be modulated by HDL. The results show that FLS contain stores of CCL2 that are released on activation by MSU crystals. Furthermore, MSU crystals also induce CCL2 gene transcription to refurbish stores. Both these MSU-crystal activities are inhibited in the presence of HDL.

## Materials and methods

### Human materials

Human synovial tissue from patients and blood from healthy volunteers was obtained with the approval of the Institutional Review Board of the University of Padova, which approved the study. An informed-consent form was signed by the patients and volunteers.

### FLS isolation and culture

Synovial tissue specimens were obtained from three osteoarthritis patients undergoing surgical joint replacement. FLS were isolated from tissue explants, as previous described [27]. In brief, synovium samples were rinsed several times in PBS, minced into ~1-mm pieces, placed in T25 flasks (Falcon, Oxnard, CA, USA), and maintained in DMEM supplemented with 10% heat-inactivated fetal calf serum (FCS), 50 μg/ml streptomycin, 50 U/ml penicillin, and 2 mmol/L glutamine (10% FCS medium). At confluence, cells were harvested (trypsin/EDTA) and seeded into new flasks. All experiments were carried out with passage 4 through 8 FLS. FLS were CD90^+^, CD55^+^, and were positive for prolyl-4-hydroxylase, as demonstrated by immunocytochemical staining with specific antibodies (Chemicon International, Temecula, CA, USA). FLS were seeded in 96-well culture plates at a density of 1 × 10^4 ^cells/well, unless otherwise stated. Cells were allowed to adhere for 24 hours, and then the medium was exchanged for a medium supplemented with 2% FCS and the indicated concentration of MSU crystals and HDL. Cell viability was assayed with trypan blue exclusion staining and was found to be higher than 98% in basal conditions.

### Isolation of HDL

Human serum HDL were isolated, and their protein content quantified, as previously described [23].

### Synthesis of MSU crystals

MSU crystals were prepared as described by Denko and Whitehouse [28]. In brief, 4 g uric acid was dissolved in 800 ml of deionized water, heated to 60°C, adjusted to pH 8.9 with 0.5 N NaOH, and let crystallize overnight at room temperature. MSU crystals were recovered by centrifugation, washed with distilled water and dried at 40°C for 24 hours. Crystal shape and birefringence were assessed by compensated polarized light microscopy. MSU crystals were milled and then sterilized by heating at 180°C for 2 hours before each experiment. Less than 0.015 EU/ml endotoxin were measured in MSU crystal preparations by Limulus amebocyte lysate assay (E-toxate kit, Sigma-Aldrich S.r.l., Milano, Italy).

### Cytokines production

FLS were stimulated by MSU crystals in DMEM supplemented with 2% heat-inactivated FCS, 50 μg/ml streptomycin, 50 U/ml penicillin, 2 mmol/L glutamine, 5 μg/ml polymyxin, and filtered before the use (2% FCS medium). HDL were added with or 1 hour before stimulation by MSU crystals. Culture supernatants were harvested and stored at -20°C before CCL2, IL-8, and IL-1β measurements by enzyme immunoassay (RayBiotech, Inc., Norcross, GA, USA). Cytotoxicity of MSU crystals and HDL was assessed with a colorimetric assay for cell proliferation and activity (MTT, Chemicon International, Temecula, CA, USA), which measures mitochondrial activity of cells. In some experiments, cells were pretreated with 10 μg/ml cycloheximide (CHX; Sigma-Aldrich S.r.l., Milano, Italy) for 30 minutes before stimulation. Alternatively, FLS were stimulated by IL-1β (Recombinant Human IL-1β; R&D systems, Minneapolis, MN, USA). Inhibition experiments were carried out with IL-1-receptor antagonist (IL-1Ra, R&D Systems, Minneapolis, MN, USA).

### Confocal microscopy

FLS were grown in eight-well chamber slides at a density of 2 × 10^4 ^cells/well in 10% FCS medium and then were incubated with MSU crystals (50 μg/ml) or HDL (50 and 100 μg/ml) or both for 24 hours in 2% FCS medium. Cells were washed 3 times in PBS, fixed with 4% paraformaldehyde for 10 minutes, and permeabilized with 0.1% Triton X-100 in PBS for 4 minutes at 4°C. After washing and blocking with 2% BSA for 30 minutes at room temperature, cells were incubated for 1 hour with anti-CCL2 mAb (R&D systems, Minneapolis, MN, USA) diluted 1:20 in blocking buffer. After washing, bound antibodies were detected by using Alexa Fluor 488-conjugated goat anti-mouse IgG secondary Ab (1:150; Invitrogen S.R.L., San Giuliano Milanese, Italy) for 30 minutes at room temperature in the dark. Samples were analyzed with confocal microscopy (2100 Multiphoton; Bio-Rad Laboratories, Inc., Italy), by using laser excitation at 488 nm.

### Chemotaxis assay

Mononuclear cells were isolated from peripheral blood from healthy volunteers by density gradient centrifugation with Histopaque 1077 (Sigma-Aldrich S.r.l., Milano, Italy). The effects of MSU crystals and HDL on the chemotaxis of mononuclear cells were assessed by using a 48-well modified Boyden chamber (AC48; NeuroProbe, Bethesda, MD, USA). Culture supernatants of FLS stimulated by MSU crystals in the presence or absence of HDL were loaded in the bottom chamber, and mononuclear cells were added to the top chamber. DMEM was used as a negative control, and 10 ng/ml CCL2 (RayBiotech, Inc., Norcross, GA, USA) was used as a positive control. A polyvinylpyrrolidone-free polycarbonate 8-mm membrane with 5-μm pores, pretreated with 10 μg/ml fibronectin, was placed between the chambers. In brief, 28-μl aliquots of culture supernatants were dispensed into the bottom wells of the chamber. Fifty-microliter aliquots of mononuclear cells (1 × 10^6 ^cells/ml) resuspended in RPMI 1640 were added to the top wells. Chambers were incubated at 37°C with 5% CO_2 _for 2 hours. The membrane was then removed, washed with PBS on the upper side, fixed, and stained with DiffQuik (Baxter Scientific, Miami, FL, USA). Cells were counted microscopically at ×1,000 magnification in four fields per membrane. All assays were performed in duplicate.

### CCL2 mRNA

FLS were grown to confluence in six-well culture dishes in 10% FCS medium and then incubated with MSU crystals (50 μg/ml) in the presence or absence of HDL (100 μg/ml) for the indicated time periods in 2% FCS medium. Supernatants were harvested for CCL2 measurements, and total FLS RNA was prepared by Tri?Reagent, as described by the provider (Sigma-Aldrich S.r.l., Milano, Italy). Quantitative real-time duplex PCR analysis (TaqMan quantitative ABI PRISM 7300 Detection System, Applied Biosystems) was conducted after reverse transcription by SuperScript II (Invitrogen S.R.L., San Giuliano Milanese, Italy). The levels of mRNA expression were normalized, with the expression of a housekeeping gene (18S) analyzed simultaneously. CCL2 and 18S probes were purchased from Applied Biosystems. All measurements were conducted in triplicate.

### Statistical analysis

When required, data significance was assessed with Student's *t *test; *P *< 0.05 was considered significant.

## Results

### MSU crystals induce CCL2 release by human FLS

**Figure 1:**
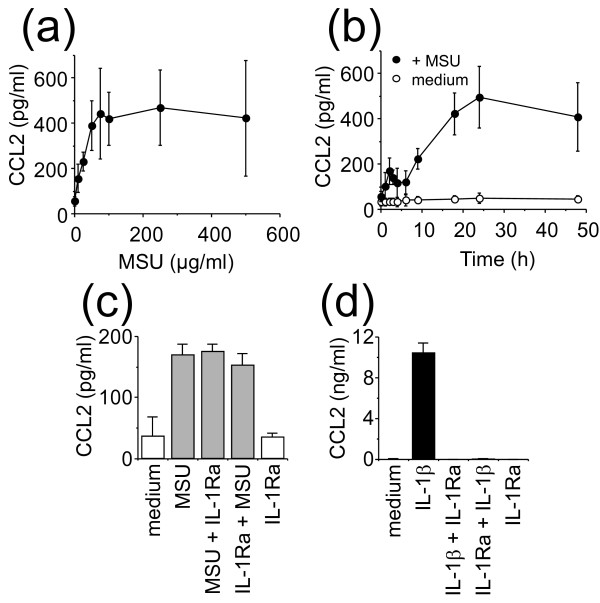
**Effect of monosodium urate (MSU) crystals on CCL2 production in cultured fibroblast-like synoviocytes (FLS)**. **(a) **FLS were treated with increasing concentrations of MSU crystals for 24 hours. **(b) **Synoviocytes were incubated in the presence (black circles) or absence (white circles) of 50 μg/ml MSU crystals for the indicated time. CCL2 was measured in culture supernatants with ELISA. Results are presented as mean ± SD of three separate experiments. **(c) **FLS were stimulated (grey columns) or not (white columns) for 24 hours with 50 μg/ml MSU crystals in the presence or absence of 250 ng/ml IL-1Ra. IL-1Ra was either added with MSU crystals (MSU + IL-1Ra) or added to FLS 1 hour before activation by MSU crystals (IL-1Ra + MSU). **(d) **FLS were stimulated (black columns) or not (white columns) for 24 hours with 125 pg/ml IL-1β in the presence or absence of 250 ng/ml of IL-1Ra. IL-1Ra was either added with IL-1β (IL-1β + IL-1Ra) or added to FLS 1 hour before activation by IL-1β (IL-1Ra + IL-1β). **(c, d) **Culture supernatants were analyzed for the production of CCL2. Results are presented as mean ± SD of three separate experiments.

To evaluate the capacity of MSU crystals to induce CCL2 release, FLS were incubated for 24 hours with increasing concentrations of MSU crystals. In the range of concentrations used, MSU crystals did not significantly affect cell viability, which was only slightly decreased at concentrations higher than 50 μg/ml (data not shown). In the absence of stimulus, FLS released low but significant levels of CCL2 amounting to 55 ± 20 pg/ml (Figure 1a). A similar pattern of CCL2 production was observed among FLS preparations, independent of the donor or cell passage, although the extent of CCL2 production varied between experiments, as indicated by the error bar dimension (Figure 1). MSU crystals induced a dose-dependent increase of CCL2 production in FLS, reaching a plateau at 50 to 75 μg/ml MSU crystals (Figure 1a). To determine the time required for maximal chemokine production, FLS were exposed to 50 μg/ml MSU crystals for increasing time periods. As depicted in Figure 1b, CCL2 production reached a plateau at 20 to 24 hours. Therefore, in the experiments described later, FLS were activated for 24 hours with an optimal dose of 50 μg/ml of MSU crystals. Because FLS might release IL-1, a potent stimulus of fibroblasts, FLS were stimulated by MSU crystals in the presence of the IL-1-specific inhibitor, IL-1Ra [29]. As shown in Figure 1c, the production of CCL2 induced by MSU crystals was not affected by 250 ng/ml IL-1Ra. In the same experiments, such an IL-1Ra dose abolished CCL2 production induced by 125 pg/ml IL-1β (Figure 1d). IL-1Ra *per se *had no effect on CCL2 production by FLS (Figure 1c and 1d). These results demonstrate that the production of CCL2 was directly induced by MSU crystals, ruling out the participation of an autocrine loop of IL-1.

### CCL2 is contained in small vesicles in FLS

**Figure 2:**
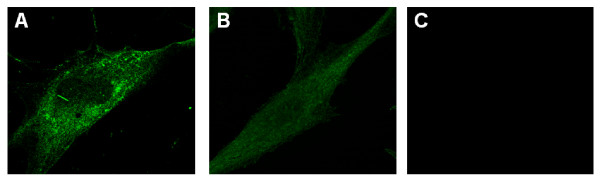
**CCL2 is stored in cytoplasmic vesicles in fibroblast-like synoviocytes (FLS)**. FLS were cultured for 24 hours in the absence **(a, c) **or presence **(b) **of 50 μg/ml monosodium urate (MSU) crystals. Cells were subjected to immunostaining with anti-CCL2 antibodies **(a, b) **or incubated with the second antibody only as a negative control **(c)**, and then analyzed with confocal microscopy, as described in Materials and Methods. Original magnification, ×1,000.

Because CCL2 is constitutively contained in small storage granules within endothelial cell cytoplasm [30,31], the presence of such a compartment was assessed in FLS with confocal microscopy. In resting FLS, CCL2 was localized in small vesicles in cell cytoplasm (Figure 2a). Consistent with CCL2 release, after 24-hour stimulation with 50 μg/ml MSU crystals, a marked diminution of the number of CCL2-containing vesicles was observed as compared with unstimulated cells (Figure 2b). These data suggest that FLS contained intracellular pools of CCL2 that was stored in small vesicles and thus might be rapidly released on stimulation.

### HDL inhibit MSU crystal-induced CCL2 production in FLS

**Figure 3:**
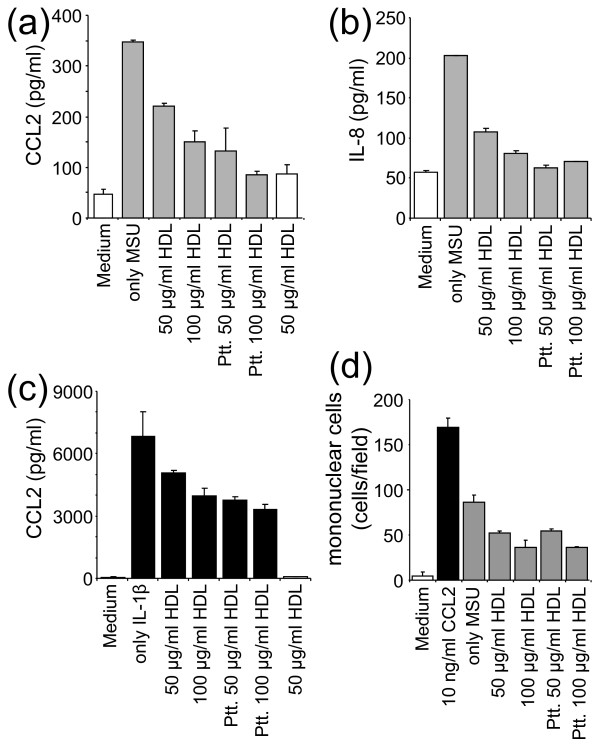
**High-density lipoproteins (HDL) inhibit CCL2 production induced by monosodium urate (MSU) crystals in fibroblast-like synoviocytes (FLS)**. **(a, b) **FLS were stimulated (grey columns) or not (white columns) for 24 hours with 50 μg/ml MSU crystals in the presence or absence of the indicated concentration of HDL. Alternatively, FLS were pretreated (Ptt.) with the indicated concentration of HDL, washed, and then stimulated for 24 hours with 50 μg/ml MSU crystals. Culture supernatants were analyzed for the production of CCL2 (a) and IL-8 (b). Results are presented as mean ± SD of duplicate determinations and are representative of independent experiments carried out with FLS isolated from three different patients. **(c) **FLS were stimulated (black columns) or not (white columns) with 10 pg/ml IL-1β for 24 hours. Culture supernatants were analyzed for the production of CCL2. **(d) **Culture supernatants of FLS, activated as in (a) and (b), were analyzed for their ability to induce mononuclear cell migration, as described in Materials and Methods. Migration induced by culture medium (white column), 10 ng/ml CCL2 (black column), and culture supernatants of FLS activated as in (a) and (b) (grey columns). Four fields were counted for the number of migrated cells. Results represent the mean ± SD of the number of cells/field in four fields. A representative experiment of three is presented.

To assess the antiinflammatory activity of HDL, FLS were incubated with MSU crystals in the presence or absence of 50 or 100 μg/ml HDL for 24 hours. As shown in Figure 3a, HDL significantly decreased the MSU crystal-induced CCL2 production in a dose-dependent manner. This inhibition was not due to the formation of complexes between HDL and MSU crystals, because similar results were obtained when FLS were pretreated with HDL before activation by MSU crystals (Figure 3a, Ptt.). In the latter setting, the inhibition of CCL2 production tended to be more pronounced when FLS were pretreated with HDL. To confirm that HDL directly affected the FLS stimulation state, as suggested by results of Figure 3a, we sought to assess the production of other cytokines and to test another FLS stimulus. As shown in Figure 3b, the MSU crystals-induced production of IL-8 was inhibited in the presence of HDL and abolished when FLS were pretreated with HDL. When FLS were activated by IL-1β, the induced CCL2 production was inhibited in the presence of HDL in a dose-dependent manner, the inhibition being more pronounced when cells were pretreated with HDL (Figure 3c). IL-1β was not detectable in MSU crystals-activated FLS supernatants (data not shown), thus ruling out a part of an autocrine loop of IL-1β, the induction of CCL2 or IL-8 production. Together, these results establish that HDL directly affected the FLS activation state. The amount of CCL2 induced by MSU crystals was very low as compared with that induced by IL-1β. However, CCL2 concentrations in supernatants of MSU crystals-activated FLS were sufficient to induce mononuclear cells migration. This effect was reduced when FLS were treated or pretreated with HDL (Figure 3d).

**Figure 4:**
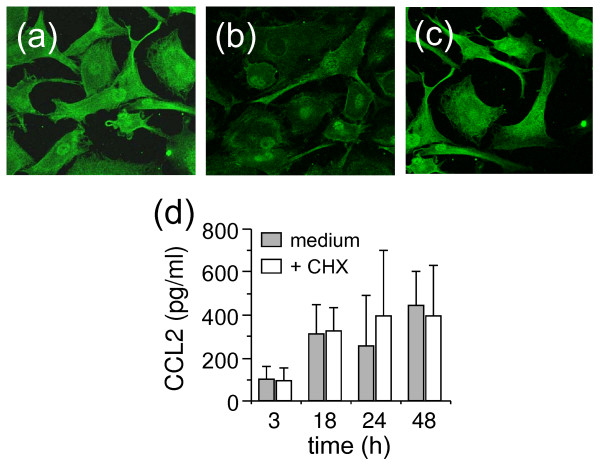
**Fibroblast-like synoviocytes (FLS) stimulated by monosodium urate (MSU) crystals in the presence of high-density lipoproteins (HDL) retain intracellular CCL2**. FLS were stimulated or not with MSU crystals (50 μg/ml) in the presence or absence of the indicated concentration of HDL. After 24 hours, cells were fixed and subjected to immunostaining with anti-CCL2 antibodies. **(a) **Resting FLS; **(b, c) **FLS stimulated with MSU crystals in the absence **(b) **or presence of HDL **(c)**. Original magnification × 600. **(d) **FLS were treated (white columns) or not (grey columns) with 10 μg/ml cycloheximide (CHX) for 30 minutes and then activated for the indicated time with 50 μg/ml MSU crystals. Results represent mean ± SD of three independent experiments.

The premise that HDL inhibited MSU crystal-induced CCL2 release by FLS was further confirmed by fluorescent microscopy. As shown in Figure 4, intracellular CCL2 was drastically diminished in FLS after activation by MSU crystals (Figure 4b), as compared with resting FLS (Figure 4a). When FLS were activated by MSU crystals in the presence of HDL, their fluorescence intensity remained similar to that of resting FLS (Figure 4a and 4c). To ascertain that all CCL2 recovered in FLS supernatants was provided by intracellular stores, the effect of cycloheximide (CHX; that is, an inhibitor of protein synthesis) was tested. As shown in Figure 4d, CHX did not affect the production of CCL2 induced by MSU crystals, at least for a period of 48 hours, demonstrating that protein neosynthesis was not required for optimal CCL2 release. This strengthened the premise that CCL2 release was a direct effect of MSU crystals and not due to an autocrine loop after the synthesis and secretion of a putative cytokine. Together, these results demonstrate that MSU crystal-activated FLS release CCL2 from cytoplasm stores, and that this release is inhibited in the presence of HDL.

### MSU crystals induce CCL2 gene transcription, which is inhibited in the presence of HDL

**Figure 5:**
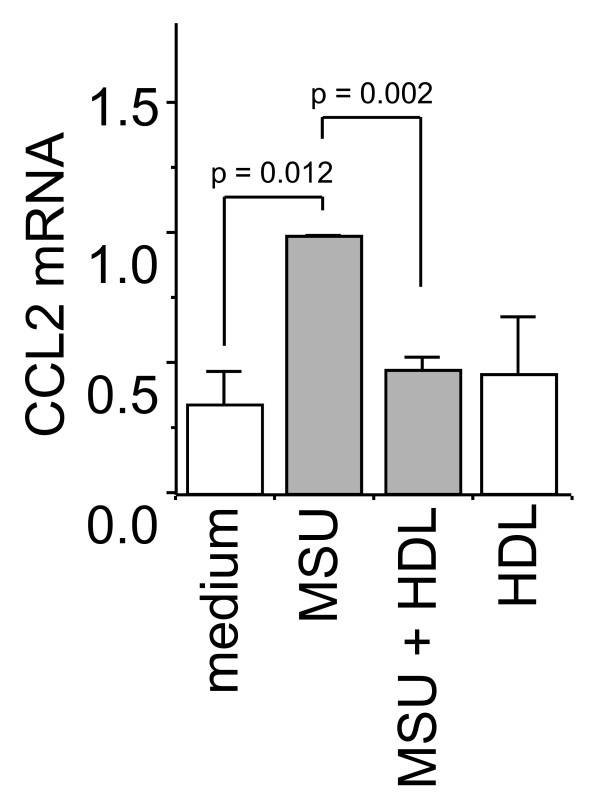
**High-density lipoproteins (HDL) diminish monosodium urate (MSU) crystal-induced CCL2 transcript levels**. Fibroblast-like synoviocytes (FLS) were cultured for 18 hours alone or with MSU crystals (50 μg/ml) in the presence or absence of HDL (100 μg/ml), as indicated. Total RNA was prepared as described in Materials and Methods. CCL2 mRNA levels were determined with duplex quantitative real-time polymerase chain reaction (PCR) analysis of triplicates normalized to the levels of the 18S mRNA. The relative expression levels of CCL2 mRNA are presented as mean ± SD of the percentage of relative CCL2 mRNA expression. The value of mRNA levels in MSU crystal-stimulated FLS (MSU) is arbitrarily considered as 1.0. Results are representative of three distinct experiments.

Because MSU crystals induced the release of CCL2, it was important to assess whether cell stimulation induced CCL2 neosynthesis (that is, increased CCL2 mRNA levels), to replenish intracellular stores after activation. To investigate the effects of MSU crystals on CCL2 mRNA levels, FLS were incubated with MSU crystals, and CCL2 transcript levels were evaluated with real-time quantitative PCR. The induction of CCL2 gene transcription in FLS activated by MSU crystals was already detectable after 2-hour stimulation and reached a maximum at 18 hours, with enhancements varying between 3 and 13 times basal levels, depending on the experiment (not shown). MSU crystal-induced expression of CCL2 mRNA was inhibited in FLS stimulated in the presence of HDL (Figure 5). In the absence of stimulus, HDL did not affect CCL2 mRNA levels. These results suggest that HDL directly acted on FLS to diminish MSU crystal-induced CCL2 production by inhibiting the release of vesicle content and by diminishing the neosynthesis of the chemokine.

## Discussion

This study demonstrates that FLS contain intracellular stores of CCL2 that are released on activation by MSU crystals. CCL2 release is accompanied by the induction of gene transcription, suggesting that MSU crystals might also trigger CCL2 store refill. Both these processes are inhibited in the presence of HDL, confirming their antiinflammatory activities and the part they might play in gout-attack limitation.

MSU crystals directly induced CCL2 production in FLS. Indeed, although MSU crystals were shown to activate the NALP3 inflammasome in mononuclear cells, resulting in IL-1β production [15], this mechanism does not apply to FLS in which MSU crystals activation does not induce the release of IL-1β. In addition to the premise that protein neosynthesis is not required for CCL2 production, our results strongly suggest that the effect of MSU crystals in FLS is not mediated by an autocrine loop of IL-1.

Intracellular stores of CCL2 were previously described in endothelial cells, where it is stocked in granules different from intracellular stores of other chemokines [30,31]. Endothelial cells are known to contain small intracellular granules that may release several inflammatory factors, including CCL2, more rapidly than the content of Weibel-Palade bodies [31,32]. Our results suggest that such a process may occur in FLS. To our knowledge, it is the first time that chemokine secretory granules were observed in FLS. The premise that CCL2 is immediately available in joints subjected to attacks of inflammatory agents suggests that in gout, monocytes may precede neutrophil infiltration. This was previously suggested in the rat air-pouch model, in which monocyte/macrophage number increases as early as 2 hours after MSU crystal injection, whereas neutrophils peak at 4 and 24 hours [33]. Thus, the presence of intracellular stores of CCL2 might participate in the rapid response of joint cells to MSU crystals, attracting monocytes/macrophages into the tissue in an attempt to eliminate the inflammatory agent rapidly.

In addition to the release of CCL2 from intracellular granules, MSU crystals induced CCL2 gene transcription in human FLS. Noticeably, CCL2 mRNA transcription was slow and peaked at 18 hours, displaying a 3-fold to 13-fold increase, as compared with basal levels in resting FLS. However, the enhancement of CCL2 was not accompanied by the enhancement of granule numbers at 24 hours. Because the production of CCL2 was not enhanced after 24-hour activation, these results suggest that the CCL2 transcript is not traduced immediately, and that longer periods are required to replenish storage granules.

The antiinflammatory role of HDL has been widely described in *in vitro *as well as in *in vivo *models of atherosclerosis [34,35]. In addition, HDL-associated apo-AI display antiinflammatory effects in other inflammatory disorders in which T-cell contact-induced cytokines production in monocytes/macrophages is likely to play a part [23,36]. HDL also potently reduce radical oxygen species production induced in neutrophils on contact with stimulated T cells [37]. Recently we demonstrated that apo A-I, HDL, and total cholesterol levels are decreased in plasma, whereas apo A-I is increased in the synovial fluid of patients with inflammatory arthritis. The correlation between synovial fluid/serum apo A-I ratio and both local and systemic inflammatory indexes suggests the involvement of HDL in the synovial inflammatory process [38]. The mechanisms of HDL antiinflammatory effects were partly identified. For instance, HDL might hamper the binding of LPS to its receptor at the cell surface, as reviewed by Wu *et al*. [39]. Similarly, it is likely that HDL impede the interaction between stimulated T cells and monocytes [23]. Here we demonstrate that HDL display antiinflammatory properties in MSU crystal-induced inflammation by decreasing the production and expression of CCL2 in human FLS. Although this study does not elucidate the mechanism of HDL action, the premise that cell preincubation with HDL resulted in an increased inhibition of CCL2 production and expression suggests that HDL may act directly on FLS either by blocking putative MSU crystal receptors/sensors or by changing the threshold of FLS response to crystals. The latter hypothesis suggests that HDL could directly signal FLS, rendering them less sensitive to inflammatory agents. Apolipoproteins, either apo B or apo E, were shown to dampen crystal-induced neutrophil activation, a mechanism that might be relevant to gout-attack resolution [19,20]. Here we show that FLS activated by MSU crystals produce CCL2 and thus may attract monocytes/macrophages into the joint. Because they inhibit this process, HDL might contribute to limit a gout attack at its very beginning by acting on resident FLS, which play a major part in chronic inflammation and the destruction of joint tissues [40].

## Conclusions

The present results demonstrate that MSU crystals induce FLS to release CCL2 that is stored in vesicles in resting conditions. This mechanism is inhibited by HDL, which may limit the inflammatory process by diminishing CCL2 production and, in turn, monocytes/macrophages recruitment in joints. Although further studies are needed to identify which signal-transduction pathways are specifically involved in the activation of FLS by MSU crystals and to elucidate the mechanism of action of HDL in the limitation of crystal-induced inflammation, this study confirms the antiinflammatory functions of HDL, which might contribute to the resolution of acute gout attack.

## Abbreviations

FLS: fibroblast-like synoviocytes; HDL: high-density lipoproteins; MSU: monosodium urate.

## Competing interests

The authors declare that they have no competing interests.

## Authors' contributions

AS, DB, FO, and LP designed research and analyzed data. AS and DB wrote the paper. AS, FO, LG, PS, AP, FF, and CA performed research.
